# Early vascular toxicity after pediatric allogeneic hematopoietic stem cell transplantation

**DOI:** 10.1038/s41409-022-01607-8

**Published:** 2022-02-17

**Authors:** Lilli Leimi, Kirsi Jahnukainen, Helena Olkinuora, Seppo Meri, Kim Vettenranta

**Affiliations:** 1 University of Helsinki, Helsinki University Hospital, Children’s Hospital, and Pediatric Research Center, Helsinki, Finland; 2 NORDFERTIL Research Lab Stockholm, Department of Women’s and Children’s Health, Karolinska Institutet and University Hospital, Stockholm, Sweden; 3 Department of Bacteriology and Immunology and Translational Immunology Research Program, University of Helsinki, and Diagnostic Center, Helsinki University Hospital, Helsinki, Finland

**Keywords:** Haematological cancer, Bone marrow transplantation, Paediatrics, Haematological diseases, Immunological disorders

## Abstract

Treatment-related mortality and morbidity remain a challenge in hematopoietic stem cell transplantation (HSCT). In this retrospective, single-center study, we analyzed endothelial damage as a potential, common denominator and mechanism for the adverse effects. We evaluated the prevalence of key vascular complications and graft-versus-host disease among 122 pediatric patients with an allogeneic HSCT between 2001 and 2013. The spectrum and frequency of acute adverse events emerging ≤100 days post transplant were graded according to the CTCAE 4.03 and analyzed. We identified a total of 19/122 (15.6%) patients with vascular complications, fulfilling the criteria of capillary leak syndrome, veno-occlusive disease/sinusoidal obstruction syndrome or thrombotic microangiopathy. The patients had a poorer overall survival (77% versus 26%, *p* < 0.001). Nearly one half (56/122, 45.9%) had at least one, severe (grade 3 or 4) adverse event. Patients with vascular complications had more often edema/effusions (*p* = 0.023), thrombocytopenia (*p* = 0.001), gastrointestinal bleeding (*p* < 0.001), acute kidney injury (*p* < 0.001), ascites (*p* < 0.001) or bilirubin increase (*p* = 0.027). These endotheliopathy-related adverse events appeared early post HSCT, varied in their clinical phenotype and predicted a poor outcome. An unrelated donor but not previous exposure to leukemia or irradiation-based conditioning was identified as a risk factor for vascular complications and endotheliopathy.

## Introduction

Allogeneic hematopoietic stem cell transplantation (allo-HSCT) is a well-established therapy for hematologic and lymphoid malignancies, but also for inborn or acquired disorders of the immune or hematopoietic systems and metabolism. The overall survival (OS) has improved due to a decrease in treatment-related mortality (TRM) with improved HSCT practices, a decrease in the incidence of graft-versus-host disease (GVHD) and improved supportive care [1–3]. Yet, TRM remains a challenge. GVHD affects the epithelium, especially in the skin, gut mucosa and biliary tract, but when refractory, also appears to involve endothelial dysfunction [4, 5]. Capillary leak syndrome (CLS), treatment-related thrombotic microangiopathy (TMA) and veno-occlusive disease (VOD) appear early on post transplant and remain associated with high mortality. The vascular complications are highly variable in their clinical course rendering early detection and timely intervention a challenge [6–8].

The clinical features related to endothelial damage post HSCT depend on the localization and extent of the damage [9]. CLS involves leakage of plasma into the interstitial space resulting in hypotension, weight gain and edema unresponsive to diuretics [10]. In turn, VOD arises from an injury to the hepatic sinusoidal endothelium resulting in activation of coagulation and obstruction of the small intrahepatic venules. VOD is characterized by hepatomegaly, portal hypertension, ascites, fluid retention, weight gain (≥5%) and jaundice (hyperbilirubinemia) [6]. The classic presentation of TMA is hemolytic anemia and thrombocytopenia with concomitant acute renal and/or central nervous system impairment, but the clinical spectrum varies [11].

In this retrospective cohort study, we studied the incidence, clinical phenotype and timing of vascular complications. Our aim was to identify adverse events related to endothelial dysfunction and our ability to predict vascular complications among pediatric recipients of allo-HSCT prior to day 100 post transplant.

## Materials and methods

### Patients

A total of 218 pediatric patients underwent an allo-HSCT between January 2001 and December 2013 at the Helsinki University Children’s Hospital, Finland. A non-sequential, but unselected cohort of 122 pediatric patients was formed based on the availability of DNA-samples for both the recipient and donor at the Finnish Red Cross Blood Service (HLA-lab), Helsinki, Finland. This national cohort randomly covers 56% of the pediatric allo-HSCT patients at our hospital within the given timeframe. The key demographic and clinical data are given in Table 1. As shown, the characteristics of the study cohort reflect those of the whole allo-HSCT population.

**Table 1:** The demographics of the study cohort and all the allo-HSCT patients transplanted at the Helsinki University Children’s Hospital during 2001–2013.

Characteristics	Study cohort	All allogeneic HSCTs	*p* value
*Number of patients, n*	122	218	
*Malignant/non-malignant, n (%)*	98 (80.3)/24 (19.7)	175 (80.3)/43 (19.7)	1.000
*Male/female, n (%)*	76 (62.3)/46 (37.7)	137 (62.8)/81 (37.2)	1.000
*Age at transplantation, median/years (range, years)*	7,7 (0.5–17.2)	7,9 (0.5–18.8)	0.786
*Diagnosis, n (%)*			0.753
ALL/NHL	73 (59.8)	128 (58.7)	
AML/CML	22 (18.0)	41 (18.8)	
Sarcomas, histiocytosis	0	4 (1.8)	
SAA/FA/MDS/JMML/other hematopoietic^a^	18 (14.8)	30 (13.8)	
Immunological and metabolic disorders	9 (7.4)	15 (6.9)	
*Disease status at transplant, malignant, n (%)*			0.558
CR1/CR2/CR3 or partial remission	51 (52.0)/33 (33.7)/14 (14.3)	88 (50.3)/72 (41.1)/15 (8.6)	
*Donor type, n (%)*			0.173
Sibling	51 (41.8)	71 (32.6)	
MUD	62 (50.8)	117 (53.7)	
Cord blood	6 (4.9)	23 (10.6)	
Family/haplo	3 (2.5)	7 (3.2)	
*Conditioning, n (%)*			0.885
fTBI + MAC	98 (80.3)	179 (82.1)	
No TBI + MAC	15 (12.3)	23 (10.6)	
RIC	9 (7.4)	16 (7.3)	
*Stem cell source BM/PBSC/cord blood, n (%)*	114 (94.3)/2 (1.6)/6 (4.9)	189 (86.7)/6 (2.8)/23 (10.6)	0.168
*Relapse after HSCT yes/no, n (%)*	24 (19.7)/98 (80.3)	45 (21.3)/166 (78.7)	0.780
*Overall survival, n (%)*	84 (68.9)	141 (64.7)	0.474
*Cause of death relapse/toxicity, n (%)*	21 (55.3)/17 (44.7)	40 (56.3)/31 (43.7)	1.000

*HSCT* hematopoietic stem cell transplantation, *CR* complete remission, *MUD* matched unrelated donor, *fTBI* fractioned total body irradiation, *MAC* myeloablative conditioning, *RIC* reduced intensity conditioning, *BM* bone marrow, *PBSC* peripheral blood stem cell.

^a^No sickle cell disease patients included.

### Outcome measures

The main aim of this study was to determine the prevalence and clinical variation in acute toxicity among pediatric allo-HSCT recipients. A specific aim was to document the prevalence and appearance of key vascular complications, i.e., CLS, TMA and VOD potentially related to endothelial damage/dysfunction.

### Data collection

Data were collected retrospectively from the medical records focusing on the first 100 days post transplant. To describe the acute toxicity after pediatric allo-HSCT, we retrospectively collected all the adverse effects described in the medical records and graded them using the Common Terminology Criteria for Adverse Events (CTCAE) 4.03 with grades 3–4 designated as serious. Moderate symptoms and findings requiring further intervention (drug therapy or alterations in it) were classified as grade 2. Grade 1 conditions (clinically insignificant) were excluded for possible bias in reporting. Any given condition was reported by the highest grade detected and only once, at the time of detection. Abnormal laboratory values were included only if they led to intervention and did not fit into the normal course of allo-HSCT (e.g., low blood counts before engraftment). Four patients got two allografts, but only the first with a 100-day follow-up was included. One patient had the first allograft long before the study timeline and, thus, only the allogeneic HSCT for the secondary cancer was included. In addition, one patient had undergone both an allo- and auto-HSCT.

### Treatment characteristics and definitions

Leukemia patients were treated according to the Nordic treatment protocols Nopho ALL-92, ALL-2000 and ALL-2008, AML-93 and AML-2004 [12–15]. The cumulative chemotherapy burden was calculated by using cyclophosphamide (Cy) equivalent doses [16] for alkylating agents and doxorubicin isotoxic equivalent for anthracyclines [17].

Patients with severe aplastic anemia received Cy 120 or 200 mg/kg with or without fractionated total body irradiation (fTBI) 10 Gy. Patients with NHL, CML and JMML were treated according to pertinent international guidelines, and for conditioning, they, as well as those with a non-malignant hematopoietic disease or immunodeficiency, received Cy (100–200 mg/kg) or high dose cytarabine (36 g/m^2^) or different combinations of fludarabine (150 mg/m^2^), treosulfan (42 mg/m^2^), thiotepa (10 mg/m^2^) and etoposide (60 mg/kg) with or without fTBI 10/12 Gy. The conditioning was classified into three groups (myeloablative with or without fTBI or reduced intensity) as shown in Table 1. Additional details on the conditioning are given in Supplementary Table S1. Prophylactic defibrotide (6.25 mg/kg q. 6 h) was used with a high risk of VOD (*n* = 52; 42.6%) during the first 3 weeks post transplant. The prevention of VOD also included administration of ursodeoxycholic acid (5 mg/kg q. 12 h) for the first 30 days or beyond and was used with 40.2% (*n* = 49) of patients.

The study was approved by the Research Ethics Committee of the Helsinki University Hospital (79/13/03/03/2016, update 3082/2018).

### Graft-versus-host disease (GVHD)

Acute GVHD (aGVHD) was graded according to the Glucksberg classification [18]. GVHD prophylaxis consisted of daily cyclosporine in 80.3% (*n* = 98) of patients, 9.8% (*n* = 12) of mycophenolate mofetil, 2.5% (*n* = 3) of sirolimus and 5.7% (*n* = 7) of a combination of the above. One patient (0.8%) did not have any GVHD prophylaxis and one (0.8%) only methotrexate. ATG was used in 31 (25%) and ALG in 21 (17%) patients. In all, 42.6% (*N* = 52) of the patients needed therapy with prednisolone (2 mg/kg/day) prior to day +100 post HSCT for grade 2 (gut or liver) or 3 (skin) aGVHD.

### The vascular origin of the transplant-related complications

Early complications of vascular origin were defined as those occurring within the first 100 days post transplant and fulfilling the diagnostic criteria of a CLS, VOD/sinusoidal obstruction syndrome (SOS) or TMA, or having clinical evidence supporting the above when, in the absence of specific laboratory aberrations, the criteria (c.f. Table 2) were only partly met. TMA was diagnosed using the criteria proposed by Cho et al. [19]. In our retrospective setting, we also included clinical signs indicative of TMA, i.e., the need for antihypertensive medication, an increase (≥×1.5) in the creatinine level from the baseline, diuresis <0.5 ml/kg/h for >6 h, need for dialysis, hemorrhagic cystitis, pericardial effusion, pulmonary hypertension, seizures, severe GI bleeding, ARDS and/or persistent fever of unknown origin.

**Table 2:** Diagnostic criteria for the vascular complications.

	Diagnostic criteria	Supplemental criteria^e^
CLS (Pagliuca et al. [21])	Rapid weight gain (>3% in 24 h)	Hypoalbuminemia
	Edema (generalized edema, pericarditis, pleural effusion or ascites) requiring intensive diuretic medication	Renal insufficiency
		Hypotension
		Tachycardia
TMA (Cho et al. [19])	Normal coagulation assays^a^	Gastrointestinal bleeding
	Decrease in hemoglobin concentration	Pericardial effusion
	Thrombocytopenia^b^	Acute kidney injury
	Decrease in haptoglobin concentration^c^	Respiratory failure
	Increase in serum LDH	Neurologic symptoms (seizures)
	Schistocytes on peripheral blood smear	Pulmonary hypertension
	Increase in serum creatinine^d^	Hemorrhagic cystitis
	Negative Coombs test	Fever of unknown origin
VOD/SOS (EBMT [20])	No limitation for time of VOD onset	
	The presence of two or more of the following:	
	Unexplained consumptive and transfusion-refractory thrombocytopenia	
	Unexplained weight gain for three consecutive days despite the use of diuretics or weight gain >5% above baseline	
	Hepatomegaly	
	Ascites	
	Rising bilirubin from baseline for 3 consecutive days or bilirubin ≥2 mg/dL within 72 h	

*CLS* capillary leak syndrome, *TMA* thrombotic microangiopathy, *VOD* veno-occlusive disease, *SOS* sinusoidal obstruction syndrome, *LDH* lactate dehydrogenase.

^a^Coagulation assays include prothrombin time and activated partial thromboplastin time.

^b^Decrease <50 × 10^9^/L or a ≥50% decrease.

^c^Decrease (below the lower limit of normal).

^d^Doubling relative to patient’s pre-HSCT baseline.

^e^Clinical findings considered as supportive for the diagnosis especially in lack of all the laboratory tests needed to criteria.

The criteria and severity grading for VOD have been suggested by the EBMT [20]. CLS was characterized by its clinical features [21] also given in Table 2 and supported by hypoalbuminemia, tachycardia, hypotension and renal insufficiency.

### Statistical analysis

Statistical analyses were performed using the IBM SPSS Statistics, version 25.0, Armonk, NY, USA. Fisher’s exact and *χ*^2^-tests for categorical variables and the Mann–Whitney *U* and Kruskal–Wallis tests for continuous variables were employed for differences between the groups. Differences between the patients with endothelium-linked disorders (CLS, TMA, VOD) and others were determined by cross-tabulations. Logistic binary regression and crosstabulation were used to obtain the odds ratio (OR) for factors predictive of endothelial damage. The ROC analysis was used in validating the adverse effects contributing to endothelial damage. Out of the four patients with two allo-HSCTs during the timeframe of analysis, only the first was included. *P* value <0.05 was employed to indicate statistical significance between the observed differences.

## Results

### Patient characteristics

Key patient characteristics are presented in Table 1. A total of 65.6% (*n* = 80) had aGVHD of any grade before day 100 post transplant. Of these, 36.3% (*n* = 29) had severe, grade 3 or 4 aGVHD in at least one organ (skin, gut or liver). The OS rate was 68.9% (84/122). Of the male patients 67.1% (*n* = 51) had a male and 32.9% (*n* = 25) a female donor. A total of 47.8% (*n* = 22) of the female patients had a female and 52.2% (*n* = 24) a male donor. All the early donors and recipients were HLA-typed for at least 6 alleles, while the latest were typed for up to 16 alleles. Most of the transplantations (66/122) were performed using HLA-matched patient–donor pairs (Supplementary Table S2).

### Acute adverse events

Out of our 122 patients, 91 (75%) had at least one adverse event and 56 (45.9%) at least one severe, grade 3 or 4 adverse event. These are listed in Table 3 by category; some patients had several adverse events within the same category.

**Table 3:** Distribution and severity of adverse events in the pediatric allo-HSCT cohort of 122 patients ≤100 days post transplant graded according to CTCAE 4.03.

System	Grade 2–4 *N* (%)	Grade 3 or 4 *N* (%)
*All*	306	130
*Auditory*	2 (2)	1 (1)
*Cardiac*		
Hypertension/hypotension	17 (14)	7 (6)
Left ventricular dysfunction	1 (1)	1 (1)
Pericardial effusion	3 (2)	0
*Dental/oral*	18 (15)	11 (9)
*Dermatology/skin*	7 (6)	3 (2)
*Gastrointestinal*		
Diarrhea	24 (20)	11 (9)
Feeding problems/anorexia	10 (8)	5 (4)
GI bleeding	9 (7)	6 (5)
Nausea/vomiting	11 (9)	2 (2)
*Hematological*	17 (14)	12 (10)
*General conditions*		
Edema, weight gain	18 (15)	2 (2)
FUO (prolonged)	14 (11)	0
Pain	14 (11)	6 (5)
Weight loss	5 (4)	1 (1)
*Hepatic*		
Ascites	5 (4)	3 (2)
Hepatic dysfunction	25 (20)	15 (12)
*Infections*		
Local	14 (11)	2 (2)
General	8 (6)	3 (2)
Sepsis	10 (8)	10 (8)
*Neurology*		
Ataxia	2 (2)	1 (1)
Peripheral neuropathy/radiculitis	4 (3)	3 (2)
PRES	3 (2)	3 (2)
Seizures	3 (2)	1(1)
*Ocular*	2 (2)	0
*Psychiatric*		
Anxiety, confusion	3 (2)	0
Depression/exhaustion	6 (5)	2 (2)
*Pulmonary*	10 (8)	5 (4)
*Renal/urinary tract*		
Acute kidney injury	24 (20)	11 (9)
Hydronephrosis	1 (1)	1 (1)
Hematuria	14 (11)	1 (1)
*Vascular*	2 (2)	1 (1)

*FUO* fever of unknown origin, *PRES* posterior reversible encephalopathy syndrome.

Three patients died because of grade 5 adverse events including two multi-organ failures and one ARDS. Five were admitted to the pediatric intensive care unit (PICU), one twice. Reasons for the PICU admissions were gastrointestinal bleeding (*n* = 3, 60%), respiratory (*n* = 1, 20%) and multi-organ failure (*n* = 1, 20%). Three patients (60%) admitted to the PICU died.

**Fig. 1:**
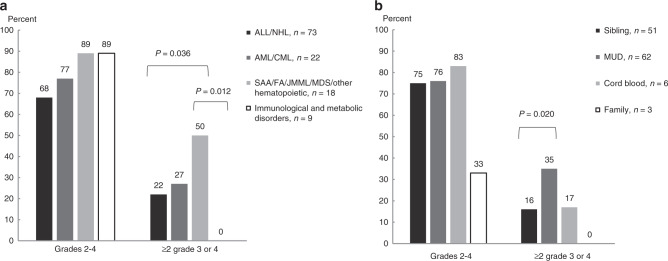
Adverse events among the 122 patients ≤100 days after pediatric allo-HSCT. Cumulative incidence of acute adverse events **a** by diagnosis and **b** donor types. Total group sizes (*n*) and significant *p* values are given.

Severe (grade 3–4) adverse events were more frequent among the females as well as those with aplastic anemia, JMML, MDS or the non-malignant, hematological diagnoses when compared to patients with immunological defects or ALL (Fig. 1a and Supplementary Table S3). Severe adverse events were more frequent also after transplantation when a matched unrelated (MUD) as opposed to a sibling donor was used (Fig. 1b and Supplementary Table S3) and among patients with severe grade 3–4 aGVHD in any target organ (Supplementary Table S3). There was no association between the number of adverse events and aGVHD (*p* = 0.518). No differences in the total number of adverse events (grade 2–4) were observed in any of the studied groups (Fig. 1a, b).

### Vascular complications

We identified a total of 19/122 (15.6%) patients with vascular complications and fulfilling the criteria of CLS, VOD/SOS or TMA. Patients with vascular complications had more MUD than sibling donors (*p* = 0.009). The OS was poorer in the group with vascular complications compared to those without (77% vs. 26%; *p* < 0.001). Median time to death varied markedly but did not differ between the groups of endotheliopathy patients and those without (145.5 [range 8–1367], *n* = 14 and 246 [65–1861] days, *n* = 24, respectively). Altogether 12/19 patients (63.2%) with vascular complications died because of treatment-related toxicity and 2 (10.5%) due to relapse. Yet, more patients (79%) without a vascular complication died because of relapse (*p* < 0.001) (Table 4). The logistic binary regression analysis and crosstabulation both demonstrated a MUD to increase the risk of a vascular complication compared to a sibling donor (OR 5.11, 95% CI 1.4–18.8, *p* = 0.014). Female gender, myeloablative conditioning, TBI, aGVHD, or a non-malignant diagnosis were not identified as risk factors. In the non-TBI group, there were three patients who received busulfan-based conditioning. Two of these had endotheliopathy, both having CLS following TMA/VOD. Endotheliopathy patients received only supportive treatment as defibrotide was already given as prophylaxis.

**Table 4:** Demographics of the patients with endothelial damage (*N* = 19) vs. those without (*N* = 103) (crosstabulation and Fisher’s exact test).

	Endothelial damage *N* (%)	Others *N* (%)	*p* value
*Gender*			NS
Male	8 (42)	68 (66)	
Female	11 (58)	35 (34)	
*Disease*			NS
Non-malignant	3 (16)	21 (20)	
Malignant	16 (84)	81 (80)	
*Diagnosis*			NS
ALL/NHL	15 (79)	58 (56)	
AML/CML	1 (5)	21 (20)	
SAA/FA/JMML/MDS/other hematopoietic^a^	2 (11)	16 (16)	
Immunological and metabolic disorders	1 (5)	8 (8)	
*Donor*			0.039
Sibling	3 (16)	48 (47)	0.009
MUD	15 (79)	47 (46)	
Cord blood	1 (5)	5 (5)	
Family/haplo	0	3 (3)	
*fTBI*			NS
No	5 (26)	19 (18)	
Yes	14 (74)	84 (82)	
*Conditioning*			NS
fTBI+MAC	14 (74)	84 (82)	
No TBI+MAC	2 (10)	12 (12)	
RIC	3 (16)	7 (7)	
*Disease status at HSCT*			NS
Non-malignant	3 (16)	21 (20)	
CR1	7 (37)	45 (44)	
CR2	7 (37)	25 (24)	
CR3 or partial remission	2 (10)	12 (12)	
*aGVHD*			NS
No	5 (26)	37 (36)	
Yes	14 (74)	66 (64)	
*Severe, grade 3–4 aGVHD*			NS
No	12 (63)	81 (79)	
Yes	7 (37)	22 (21)	
*Survival*			<0.001
Yes	5 (26)	79 (77)	
No	14 (74)	24 (23)	
*Cause of death*			<0.001
Relapse	2 (14)	19 (79)	
Toxic	12 (86)	5 (21)	

*MUD* matched unrelated donor, *fTBI* fractionated total body irradiation, *MAC* myeloablative conditioning, *RIC* reduced intensity conditioning, *CR* complete remission, *aGVHD* acute GVHD.

^a^No sickle cell disease patients included.

Patients with vascular complications showed a significantly higher frequency of edema/effusions (10.5% vs 0, *p* = 0.023), thrombocytopenia (32% vs 4%, *p* = 0.001), gastrointestinal bleeding (26% vs 1%, *p* < 0.001), acute kidney injury (32% vs 2%, *p* < 0.001), ascites (21% vs 0, *p* < 0.001) and bilirubin increase (16% vs 2%, *p* = 0.027) of grades 3–4 than those without, respectively. We used the ROC analysis to assess how well any of these six adverse events could identify the key vascular complications.

**Fig. 2:**
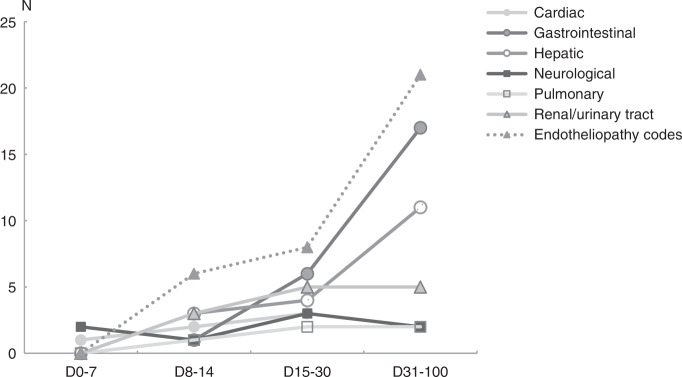
The timeline of the appearance of acute, grade 3 or 4 toxicity after pediatric allogeneic HSCT in six key organ systems. Included is also the appearance of the six endotheliopathy codes as a group.

The AUC was 0.87 showing that the detection of any of the six had good predictability as a group with a sensitivity of 78.9% and a false positive rate (1–specificity) of only 8.7%, when using one event as the cut-off. The six endotheliopathy-related adverse events showed early presentation when compared to other categories of toxicity (Fig. 2).

Finally, we determined the timeline for the key vascular complications. Patients with symptoms indicating capillary leak presented during the first 2 weeks post transplant. These included rapid weight gain without infection or other reasons, need for diuretics, hypoalbuminemia and effusions. Out of 13 patients who had CLS, eight had it just before or during the engraftment, one a week and two more than 3 weeks before engraftment, one a week after engraftment and a further one with CLS died before engraftment. The TMA-like symptoms appeared around 30 days post transplant with or without a previous CLS. These included a prolonged need for platelet and red blood cell transfusions, red cell fragmentation, acute kidney injury, severe gastrointestinal bleeding and hematuria. The TMA-patients also showed toxic epidermal necrolysis (*n* = 2), peripheral neuropathy (*n* = 2), neurological symptoms/seizures (*n* = 3) and sensorineural hearing loss (*n* = 2). Most of the patients had adverse events related to TMA or a hybrid TMA/VOD (15/19, 79%) with only one having a classical VOD without events related to TMA. One had capillary leak followed by aGVHD and two recovered after CLS without continuing on a path to “progressive endothelial damage”.

## Discussion

Treatment-related toxicity remains a significant, clinical problem even in pediatric allo-HSCT [22]. Our national, single-center study shows that 75% of the patients had at least one, grade 2–4 toxic event prior to day 100 post transplant. One half of the observed adverse events were severe. We identified a total of 19 patients with clinical signs of the key vascular/endothelial complications (CLS, TMA, TMA/VOD, VOD). We further identified a total of six adverse events significantly predictive of these vascular complications. Endotheliopathy-related adverse events presented early. The capillary leak associated events appeared first followed by VOD and TMA. The identification of any of the six key, endotheliopathy-related adverse events predicted vascular complications with good sensitivity but poor specificity.

Our study shows injury to the vascular endothelium to be relatively common following allo-HSCT in children. Endotheliopathy, emerging as a common denominator in the pathogenesis of multiple complications, can be caused by hitherto insufficiently understood underlying mechanisms. An immune origin or incompatibility between the recipient and donor is suggested by the five-fold more common occurrence after transplantation from unrelated donors.

Many risk factors for vascular complications have been postulated [23–27]. Yet, the definition of clear-cut clinical entities has remained challenging. In our study we managed to define six key adverse events defining patients with endothelial dysfunction. All of these remain frequent post HSCT as such but, when severe, seem to indicate endothelial damage.

Recipient genetics have been postulated to predispose to endothelium-related problems [28, 29]. The vascular endothelium protects tissues from potentially harmful, circulating impacts. Yet, the endothelium also needs to protect itself from the potentially detrimental effects of both the innate and adaptive immune systems. We hypothesize that, in addition to the GvH reactivity, non-HLA incompatibility between the host and the donor may play a role.

The incidence of vascular complications in our study was 15.6% (19/122) compared to the 10–30% reported for TMA [30–33]. Yet, the incidence of VOD was notably lower than the 8–14% reported [6] with our prophylactic use of defibrotide (42.6%, 52/122) most likely having an impact. The complement system, with its potentially cytotoxic and inflammation-inducing activity, may threaten the donor-derived cells, if the complement regulators on the surfaces of the cells are not compatible with complement factors in plasma. This could occur due to genetic variation or through immune responses against the regulatory molecules. Innate immune responses in blood, like complement activation, may influence the integrity of the endothelium and cause damage resembling TMA, in which chronic complement activation damages the endothelium thus promoting coagulation and thrombotic complications. TMA usually occurs around days 30–60 post transplant while CLS develops during the first 2 weeks [9, 21, 34, 35]. The onset of clinical symptoms indicative of endothelial damage in our study was in line with the previous studies.

The mortality of the patients with TMA has been reported high at 50–75% [25, 30]. Jodele et al. [31] reported the non-relapse mortality at 1 year post transplant to be significantly higher among those with TMA (43.6%) versus those without (7.8%). In our study, the non-relapse mortality was 13.9% (17/122) overall, but 63.2% (12/19) among those with endothelial damage (mostly TMA) and only 4.9% (5/103) among those without (*p* < 0.001). The significantly lower relapse-related mortality among those with endothelial damage may result from the same immunological mechanisms that cause both the antitumor effect and vascular events.

While the incidence of vascular complications in our study was in line with the findings of Dandoy et al. [36] (16% vs 19%), the OS was poorer. The OS among our allo-HSCT patients with endothelial damage during the first 6 months after transplant was 53% (10/19) compared to the 77% (76/98) of Dandoy et al., possibly due to the retrospective nature of our study putatively excluding patients with milder symptoms.

The limitation of our study is the size and heterogeneity of our cohort as well as its historic nature in terms of the conditioning regimens. With the CTCAE-event coding system being rather rigid even the key clinical problems may not be easily amenable to reporting. For example, problems of the same grade may not render an equal, clinical impact, with, i.e., some of those with several grade 2 problems ending up with a severe, overall toxic burden. The strength of our study is the uniform treatment of the primary malignant disease using the Nordic treatment protocols and the centralization of more than 95% of the pediatric HSCTs in Finland to one, single center. Thus, our study cohort represents well a national, pediatric allo-HSCT population within the given timeframe.

The purpose of the current study was to explore the complications among children following HSCT and analyze their potential relationship to vascular pathology. Problems related to endothelial damage affect treatment-related morbidity and mortality in pediatric HSCT. Outside the established diagnostic criteria of TMA and VOD stays a broader entity of “endotheliopathy” often recognized by the clinicians but not easily amenable to classification. Thus, several, important questions can be posed. Why do some patients get serious problems and others not? Are endothelial problems arising from a “double hit” with a patient with a relevant genetic vulnerability and exposure to toxic treatments developing endothelial damage? Is a clinical “endotheliopathy” as the phenotype potentially amenable to pharmacologic intervention with, e.g., defibrotide or eculizumab. This could, possibly, be modeled by pre-eclampsia with an established complement dysregulation [37] and the action of two different immune systems, complement activators and inhibitors, as in HSCT. Therapy prior to the HSCT is also likely to predispose the patient to endothelial damage, coagulation and complement cascade activation. In our study, however, exposure to previous leukemia therapy or myeloablative conditioning with total body irradiation was not identified as risk factor for vascular complications and endotheliopathy. Appropriate genotyping of both the donor and recipient may, in the future, render us with tools to identify early on those susceptible to endothelial damage during the allo-HSCT process.

Treatment-related toxicity is a significant clinical problem in pediatric allo-HSCT. Injury to the vascular endothelium is relatively common after allo-HSCT with different clinical phenotypes suggesting yet unidentified underlying mechanisms. The fact that a MUD increased the risk of vascular complications five-fold is suggestive of additional immune mechanisms playing a role. Recognition of these would enable a targeted follow-up of the high-risk patients and likely impact their long-term survival.

## Supplementary information

Supplementary Table S1.

Supplementary Table S2.

Supplementary Table S3.

## References

[1] Gooley TA, Chien JW, Pergam SA, Hingorani S, Sorror ML, Boeckh M (2010). Reduced mortality after allogeneic hematopoietic-cell transplantation. N Engl J Med.

[2] Brissot E, Rialland F, Cahu X, Strullu M, Corradini N, Thomas C (2016). Improvement of overall survival after allogeneic hematopoietic stem cell transplantation for children and adolescents: a three-decade experience of a single institution. Bone Marrow Transplant.

[3] Mateos MK, O’Brien TA, Oswald C, Gabriel M, Ziegler DS, Cohn RJ (2013). Transplant-related mortality following allogeneic hematopoeitic stem cell transplantation for pediatric acute lymphoblastic leukemia: 25-year retrospective review. Pediatr Blood Cancer.

[4] Zeisbrich M, Becker N, Benner A, Radujkovic A, Schmitt K, Beimler J (2017). Transplant-associated thrombotic microangiopathy is an endothelial complication associated with refractoriness of acute GvHD. Bone Marrow Transplant.

[5] Luft T, Dietrich S, Falk C, Conzelmann M, Hess M, Benner A (2011). Steroid-refractory GVHD: T-cell attack within a vulnerable endothelial system. Blood.

[6] Dalle JH, Giralt SA (2016). Hepatic veno-occlusive disease after hematopoietic stem cell transplantation: risk factors and stratification, prophylaxis, and treatment. Biol Blood Marrow Transplant.

[7] Jodele S, Davies SM, Lane A, Khoury J, Dandoy C, Goebel J (2014). Diagnostic and risk criteria for HSCT-associated thrombotic microangiopathy: a study in children and young adults. Blood.

[8] Jodele S, Dandoy CE, Myers KC, El-Bietar J, Nelson A, Wallace G (2016). New approaches in the diagnosis, pathophysiology, and treatment of pediatric hematopoietic stem cell transplantation-associated thrombotic microangiopathy. Transfus Apheresis Sci.

[9] Carreras E, Diaz-Ricart M (2011). The role of the endothelium in the short-term complications of hematopoietic SCT. Bone Marrow Transplant.

[10] Lucchini G, Willasch AM, Daniel J, Soerensen J, Jarisch A, Bakhtiar S (2016). Epidemiology, risk factors, and prognosis of capillary leak syndrome in pediatric recipients of stem cell transplants: a retrospective single-center cohort study. Pediatr Transplant.

[11] Ho VT, Cutler C, Carter S, Martin P, Adams R, Horowitz M (2005). Blood and Marrow Transplant Clinical Trials Network Toxicity Committee Consensus Summary: thrombotic microangiopathy after hematopoietic stem cell transplantation. Biol Blood Marrow Transplant.

[12] Schmiegelow K, Forestier E, Hellebostad M, Heyman M, Kristinsson J, Söderhäll S (2010). Long-term results of NOPHO ALL-92 and ALL-2000 studies of childhood acute lymphoblastic leukemia. Leukemia.

[13] Toft N, Birgens H, Abrahamsson J, Griškevičius L, Hallböök H, Heyman M (2018). Results of NOPHO ALL2008 treatment for patients aged 1-45 years with acute lymphoblastic leukemia. Leukemia.

[14] Lie SO, Abrahamsson J, Clausen N, Forestier E, Hasle H, Hovi L (2005). Long-term results in children with AML: NOPHO-AML Study Group – report of three consecutive trials. Leukemia.

[15] Abrahamsson J, Forestier E, Heldrup J, Jahnukainen K, Jónsson ÓG, Lausen B (2011). Response-guided induction therapy in pediatric acute myeloid leukemia with excellent remission rate. J Clin Oncol.

[16] Green DM, Nolan VG, Goodman PJ, Whitton JA, Srivastava D, Leisenring WM (2014). The cyclophosphamide equivalent dose as an approach for quantifying alkylating agent exposure: a report from the Childhood Cancer Survivor Study. Pediatr Blood Cancer.

[17] Shankar SM, Marina N, Hudson MM, Hodgson DC, Adams MJ, Landier W (2008). Monitoring for cardiovascular disease in survivors of childhood cancer: report from the Cardiovascular Disease Task Force of the Children’s Oncology Group. Pediatrics.

[18] Glucksberg H, Storb R, Fefer A, Buckner CD, Neiman PE, Clift RA (1974). Clinical manifestations of graft-versus-host disease in human recipients of marrow from HL-A-matched sibling donors. Transplantation.

[19] Cho BS, Yahng SA, Lee SE, Eom KS, Kim YJ, Kim HJ (2010). Validation of recently proposed consensus criteria for thrombotic microangiopathy after allogeneic hematopoietic stem-cell transplantation. Transplantation.

[20] Corbacioglu S, Carreras E, Ansari M, Balduzzi A, Cesaro S, Dalle JH (2018). Diagnosis and severity criteria for sinusoidal obstruction syndrome/veno-occlusive disease in pediatric patients: a new classification from the European society for blood and marrow transplantation. Bone Marrow Transplant.

[21] Pagliuca S, Michonneau D, Sicre de Fontbrune F, Sutra Del Galy A, Xhaard A, Robin M (2019). Allogeneic reactivity-mediated endothelial cell complications after HSCT: a plea for consensual definitions. Blood Adv.

[22] Nava T, Ansari M, Dalle JH, de Heredia CD, Güngör T, Trigoso E (2020). Supportive care during pediatric hematopoietic stem cell transplantation: beyond infectious diseases. A report from workshops on supportive care of the Pediatric Diseases Working Party (PDWP) of the European Society for Blood and Marrow Transplantation (EBMT). Bone Marrow Transplant.

[23] Batts ED, Lazarus HM (2007). Diagnosis and treatment of transplantation-associated thrombotic microangiopathy: real progress or are we still waiting?. Bone Marrow Transplant.

[24] Willems E, Baron F, Seidel L, Frere P, Fillet G, Beguin Y (2010). Comparison of thrombotic microangiopathy after allogeneic hematopoietic cell transplantation with high-dose or nonmyeloablative conditioning. Bone Marrow Transplant.

[25] Gavriilaki E, Sakellari I, Anagnostopoulos A, Brodsky RA (2017). Transplant-associated thrombotic microangiopathy: opening Pandora’s box. Bone marrow Transplant.

[26] Nakamae H, Yamane T, Hasegawa T, Nakamae M, Terada Y, Hagihara K (2006). Risk factor analysis for thrombotic microangiopathy after reduced-intensity or myeloablative allogeneic hematopoietic stem cell transplantation. Am J Hematol.

[27] Rosenthal J (2016). Hematopoietic cell transplantation-associated thrombotic microangiopathy: a review of pathophysiology, diagnosis, and treatment. J Blood Med.

[28] Jodele S, Zhang K, Zou F, Laskin B, Dandoy CE, Myers KC (2016). The genetic fingerprint of susceptibility for transplant-associated thrombotic microangiopathy. Blood.

[29] Jodele S, Licht C, Goebel J, Dixon BP, Zhang K, Sivakumaran TA (2013). Abnormalities in the alternative pathway of complement in children with hematopoietic stem cell transplant-associated thrombotic microangiopathy. Blood.

[30] Elsallabi O, Bhatt VR, Dhakal P, Foster KW, Tendulkar KK (2016). Hematopoietic stem cell transplant-associated thrombotic microangiopathy. Clin Appl Thrombosis/Hemost.

[31] Jodele S, Laskin BL, Dandoy CE, Myers KC, El-Bietar J, Davies SM (2015). A new paradigm: diagnosis and management of HSCT-associated thrombotic microangiopathy as multi-system endothelial injury. Blood Rev.

[32] Willems E, Baron F, Seidel L, Frère P, Fillet G, Beguin Y (2010). Comparison of thrombotic microangiopathy after allogeneic hematopoietic cell transplantation with high-dose or nonmyeloablative conditioning. Bone Marrow Transplant.

[33] Sakellari I, Gavriilaki E, Boussiou Z, Batsis I, Mallouri D, Constantinou V (2017). Transplant-associated thrombotic microangiopathy: an unresolved complication of unrelated allogeneic transplant for hematologic diseases. Hematological Oncol.

[34] Cooke KR, Jannin A, Ho V (2008). The contribution of endothelial activation and injury to end-organ toxicity following allogeneic hematopoietic stem cell transplantation. Biol Blood Marrow Transplant.

[35] Li A, Wu Q, Davis C, Kirtane KS, Pham PD, Sorror ML (2019). Transplant-associated thrombotic microangiopathy is a multifactorial disease unresponsive to immunosuppressant withdrawal. Biol Blood Marrow Transplant.

[36] Dandoy CE, Rotz S, Alonso PB, Klunk A, Desmond C, Huber J (2020). A pragmatic multi-institutional approach to understanding transplant-associated thrombotic microangiopathy after stem cell transplant. Blood Adv.

[37] Pierik E, Prins JR, van Goor H, Dekker GA, Daha MR, Seelen MAJ (2020). Dysregulation of complement activation and placental dysfunction: a potential target to treat preeclampsia?. Front Immunol.

