# Supplementary material for: Early vascular toxicity after pediatric allogeneic hematopoietic stem cell transplantation
Source: Bone Marrow Transplant. 2022 Feb 17;57(5):705–11. doi: 10.1038/s41409-022-01607-8 (PMC9090633; doi:10.1038/s41409-022-01607-8)
Supplement: Supplementary file 1 — Supplementary Table S1. [file 41409_2022_1607_MOESM1_ESM.pdf]

Supplementary Table S1. The conditioning regimens used in the pediatric allo-HSCT cohort of 122 patients.

**MAC w fTBI**	
	Cyclophosphamide 120 mg/kg or 200 mg/kg
	Cyclophosphamide 120 mg/kg, etoposide 60 mg/kg
	Cyclophosphamide 120 mg/kg, busulfan 12,8 mg/kg
	Cyclophosphamide 120 mg/kg, busulfan 12,8 mg/kg, etoposide 60 mg/kg
	Cytarabine 36 g/m2
	Cytarabine 26 g/m2, etoposide 60 mg/kg
	Fludarabin 150 mg/m2, treosulfan 42 mg/m2
	Etoposide 60 mg/kg, thiotepa 10 mg/m2
	Etoposide 60 mg/kg
**No TBI + MAC**	
	Cyclophosphamide 200 mg/kg, busulfan 8,95 mg/kg
	Cyclophosphamide 120 mg/kg, busulfan 8,95 mg/kg, melphalan 140 mg/m2
	Cyclophosphamide 120 mg/kg, fludarabin 80 mg/m2, treosulfan 42 g/m2 with or without TLI
	Cyclophosphamide 200 mg/kg, treosulfan 42 mg/m2
	Fludarabin 150 mg/m2, treosulfan 42 mg/m2
	Fludarabine 150 mg/m2, thiotepa 10 mg/m2, melphalan 140 mg/m2
	Fludarabin 150 mg/m2, thiotepa 900 mg/m2, topotecan 6 mg/m2
	Fludarabin 6 mg/kg, treosulfan 1260 mg/kg, thiotepa 10 mg/kg
**RIC**	
	Cyclophosphamide 200 mg/kg
	Cyclophosphamide 200 mg/kg, ATG

Abbreviations: fTBI = fractionated TBI, MAC = myeloablative conditioning, RIC = reduced intensity conditioning, TLI = total lymphoid irradiation, ATG = antithymocyte globulin
