# Supplementary material for: Early vascular toxicity after pediatric allogeneic hematopoietic stem cell transplantation
Source: Bone Marrow Transplant. 2022 Feb 17;57(5):705–11. doi: 10.1038/s41409-022-01607-8 (PMC9090633; doi:10.1038/s41409-022-01607-8)
Supplement: Supplementary file 2 — Supplementary Table S2. [file 41409_2022_1607_MOESM2_ESM.pdf]

Supplementary table S2. The HLA-matching groups and alleles in the pediatric allo-HSCT cohort of 122 patients.

**Group**	**N (%)**
HLA identical sibling	49 (40)
HLA identical other relative	1 (1)
HLA identical unrelated donor	16 (13)
Related or unrelated donor, 1 antigen mismatch	24 (20)
Related or unrelated donor, 2 or more antigen mismatch	26 (21)
Cord blood, matched	2 (2)
Cord blood, mismatched	4 (3)
**Alleles**	
6 alleles (A/B/DRB1)	45 (37)
8 alleles (A/B/C/DRB1)	3 (2)
10 alleles (A/B/C/DRB1/DPB1)	10 (8)
12 alleles (A/B/C/DRB1/DPB1 and DQA1 or DQB1)	6 (5)
13 alleles (A/B/C/DRB1/DQA1/DQB1 and DRB3 or DRB5 1 loci)	4 (3)
14 alleles (A/B/C/DRB1/DPB1/DQA1/DQB1)	26 (21)
15 alleles (B/C/DRB1/DPB1/DQA1/DQB1 and A/DPB1/DRB3/DRB5 1 loci)	10 (8)
16 alleles (A/B/C/DRB1/DPB1/DRB5/DQA1/DQB1)	18 (15)
