# Supplementary material for: Early vascular toxicity after pediatric allogeneic hematopoietic stem cell transplantation
Source: Bone Marrow Transplant. 2022 Feb 17;57(5):705–11. doi: 10.1038/s41409-022-01607-8 (PMC9090633; doi:10.1038/s41409-022-01607-8)
Supplement: Supplementary file 3 — Supplementary Table S3. [file 41409_2022_1607_MOESM3_ESM.pdf]

Supplementary Table S3. Number of severe grade 3 or 4 adverse events in the 122 pediatric allo-HSCT patients ≤ 100 days post-transplant. Relationships between categorical variables are examined by crosstabulation and Fisher´s Exact test.

	**0–1 grade 3 or 4 N (%)**	**≥2 grade 3 or 4 N (%)**	**p-value**
All patients	91 (75)	31 (25)	
Gender			0.032
Male	62 (68)	14 (45)	
Female	29 (32)	17 (55)	
Disease			NS
Non-malignant	17 (19)	7 (23)	
Malignant	74 (81)	24 (77)	
Diagnosis			0.029
ALL/NHL	57 (63)	16 (52)	
AML/CML	16 (18)	6 (19)	
SAA/FA/JMML/MDS/other hematopoietic	9 (10)	9 (29)	
Immunological and metabolic disorder	9 (10)	0	
Donor			NS
Sibling	43 (47)	8 (26)	] 0.020
MUD	40 (44)	22 (71)	
Cord blood	5 (6)	1 (3)	
Family/haplo	3 (3)	0	
fTBI			NS
No	16 (18)	8 (26)	
Yes	75 (82)	23 (74)	
Conditioning			NS
fTBI+MAC	75 (82)	23 (74)	
No TBI+MAC	10 (11)	7 (23)	
RIC	6 (7)	1 (3)	
aGVHD			NS
No	33 (36)	9 (29)	
Yes	58 (64)	22 (71)	
Grade 3–4 aGVHD			0.030
No	74 (81)	19 (61)	
Yes	17 (19)	12 (39)	
Defibrotide			NS
No	57 (63)	13 (42)	
Yes	34 (37)	18 (58)	
Survival			0.007
Yes	69 (76)	15 (48)	
No	22 (24)	16 (52)	

Abbreviations: fTBI = fractionated TBI, MAC = myeloablative conditioning, RIC = reduced intensity conditioning, aGVHD = acute GVHD
